# The relationship between clinicopathological variables, systemic inflammation, and CT‐derived body composition with survival in patients with advanced non‐small cell lung cancer receiving nivolumab as a second‐line treatment

**DOI:** 10.1002/cam4.6805

**Published:** 2023-12-13

**Authors:** Randa Saeed, Josh McGovern, Hugo Bench, Ross D. Dolan, Donald C. McMillan, Almudena Cascales

**Affiliations:** ^1^ Academic Unit of Surgery, School of Medicine University of Glasgow Glasgow UK; ^2^ NHS Lanarkshire Glasgow UK; ^3^ Department of Clinical Oncology Beatson West of Scotland Cancer Centre Glasgow UK

**Keywords:** body composition, ECOG‐PS, hypoalbuminemia, immunotherapy, Nivolumab, NSCLC, survival, systemic inflammation

## Abstract

**Background:**

Second‐line immunotherapy is currently recognized to help only a subset of patients with advanced forms of non‐small cell lung cancer (NSCLC). The current study analyzes the connection between prior treatment host/tumor characteristics and survival in advanced NSCLC patients receiving nivolumab as a second‐line therapy.

**Methods:**

A retrospective cohort analysis was carried out on individuals with advanced NSCLC receiving second‐line Nivolumab with palliative intent between February 2016 and May 2019 across three health boards in NHS Greater Glasgow and Clyde, Lanarkshire, Ayrshire, and Arran in Scotland to examine the association between systemic inflammation, body composition, and survival were determined using computed tomography (CT).

**Results:**

The current study investigates the connection between prior treatment host/tumor characteristics and survival in advanced NSCLC patients receiving nivolumab as a second‐line therapy. The majority were 65 years of age or older (51%), female (53%), had adenocarcinoma (53%), and had good performance status (ECOG 0/1) (86%). Most patients had high SFI (70%) or VFA (54%). The median overall survival after starting Nivolumab was 15 months. ECOG‐PS and hypoalbuminemia were significant predictors of 12‐month survival in patients with advanced NSCLC following Nivolumab treatment, according to Cox regression (*p*‐value = 0.047 and 0.014, respectively).

**Conclusion:**

In patients with advanced NSCLC receiving Nivolumab as a second‐line therapy, ECOG‐PS and hypoalbuminemia were strongly associated with survival. Systemic inflammation and hypoalbuminemia measurements may enhance the ECOG‐PS stratification of expected outcomes.

## Abstract 2

The relationship between pre‐treatment clinicopathological variables, systemic inflammation, CT‐body composition, and 12 months survival in patients with NSCLC receiving Nivolumab as a second line treatment.

## 1 INTRODUCTION

Despite declining incidence rates, lung cancer accounts for 13% of all newly diagnosed cancers in the United Kingdom (UK) and is still the third most common malignancy.^1^ It is the leading cause of cancer death in the UK, accounting for 21% of all cancer fatalities.^1^ This is partly because the majority of patients present with TNM stage III or IV illness at diagnosis and are thus treated with palliative aim.^2^ Individuals with distal metastatic illness (stage IV), for instance, had a 1‐year survival rate of just 15%–20%, in contrast to 81%–85% for stage I.^3^

Small cell lung cancer (SCLC) and non‐small cell lung cancer (NSCLC) are the two basic types of cancer of the lungs; the latter is responsible for around 85% of incidents.^4^ Long the cornerstone for patients with advanced NSCLC, chemotherapy has improved survival.^5^ However, clinical results have improved since the development of immune checkpoint inhibitors (ICI) in the previous 10 years, with a plethora of evidence demonstrating that they increase patients' overall survival in NSCLC patients compared to chemotherapy alone.^6^ However, only around 20% of NSCLC patients benefit from ICI treatment.^7^ To identify the individuals with NSCLC who may benefit most from these innovative treatment drugs, there is an ongoing interest in the association between host phenotype and clinical results in these patients receiving ICI.

Host phenotypic features such as weight loss, low body mass index (BMI), sarcopenia, and systemic inflammation have long been linked to poor outcomes in patients with advanced NSCLC.^8^, ^9^, ^10^ BMI has restrictions as it does not account for body composition factors such as muscle volume or regional fat distribution, which may have a different impact on survival.^11^ Recent research has discovered that sarcopenia and fat tissue (rather than BMI) are independent risk factors for ICI efficacy in tumors.^11^ Obesity and reduced muscle quantity and quality were linked to poor results in one trial of ICI‐treated melanoma patients.^12^ The loss of lean tissue, in particular, is a key indicator of cancer cachexia.^13^ Shiroyama and colleagues discovered that baseline sarcopenia was significantly associated with poor survival outcomes in patients with advanced non‐small cell lung cancer treated with ICIs.^14^

As a result, the current study examines the relationship between host features, including systemic inflammation and CT‐derived body composition, and survival in patients with advanced NSCLC receiving second‐line Nivolumab with palliative intent.

## 2 PATIENTS AND METHODS

All patients with advanced NSCLC who received at least one cycle of Nivolumab as a second, third, or subsequent line of therapy in three Scottish health boards (NHS Greater Glasgow and Clyde, Lanarkshire, Ayrshire, & Arran) between September 2016 and January 2019 had their data collected retrospectively. Patients were eligible for this retrospective database cohort study if they had an abdominal CT scan (3 months) and a full blood count (1 month) prior to starting Nivolumab medication. Caldicott Guardian Approval (NHS Greater Glasgow and Clyde) gave ethical approval for this audit study. This study was conducted in response to the Helsinki Declaration.^15^ The survival date was determined from the moment Nivolumab was administered to death or the censor day.

### 2.1 Clinicopathological characteristics

Before starting Nivolumab, routine demographic information about each patient was gathered. This information comprised age, sex, histology, BMI, neutrophil‐to‐lymphocyte ratio (NLR), and hypoalbuminemia. Groups based on age were >65, 65–74, and <75 years old. A medical practitioner or clinical researcher working at the facility where the patient was being treated created the Eastern Cooperative Oncology Group‐Performance Status (ECOG‐PS). Depending on their ECOG‐PS, participants were split into “0,” “1,” and “> 2” categories. Four BMI categories were “<20, 20–24.9, 25–29.9, and ≥30” kg/m^2^. NLR levels were classified as “<3, 3–5, and >5” based on the patient's total blood count; this was calculated by dividing the neutrophil count by the lymphocyte count. Less than 35 g/L of serum albumin was considered hypoalbuminemia.

### 2.2 CT body composition analysis

As previously stated, CT images were obtained at the third lumbar vertebral level.^10^ Images lacking regions of interest or exhibiting notable movement artifacts were excluded from consideration for inclusion. A freeware program called Version 1.47 of the NIH ImageJ software (http://rsbweb.nih.gov/ij/) was used to analyze each picture and was found to produce accurate measurements.^16^ Total fat area (TFA, cm^2^), visceral fat area (VFA, cm^2^), and skeletal muscle area (SMA, cm^2^) were calculated as ROIs using standard Hounsfield Unit (HU) values (adipose tissue −190 to −30, and skeletal muscle −29 to +150). The subcutaneous fat area (SFA, cm^2^) was computed by subtracting the VFA from the TFA. After normalizing the SFA and SMA for height 2, the skeletal muscle index (SMI, cm^2^/m^2^) and subcutaneous fat index (SFI, cm^2^/m^2^) were calculated. Skeletal muscle radiodensity (SMD, HU) was measured using the same ROI to compute SMI as its mean HU. These indices were then compared to preset body composition cutoff thresholds.

For males, a high SFI was defined as >50.0 cm^2^/m^2^, and for females, ≥42.0 cm^2^/m^2^ ^17^ For male patients, visceral obesity was defined as VFA >160 cm^2^, and for female patients, >80 cm^2^ ^18^ According to Martin et al.,^19^ a low SMI was defined as follows: <43 cm^2^/m^2^ if BMI <25 kg/m^2^, <53 cm^2^/m^2^ if BMI ≥25 kg/m^2^ in patients who were male, and <41 cm^2^/m^2^ in patients who were female if the BMI < or ≥ 25 kg/m^2^. In individuals with a BMI < 25 kg^2^/m^2^ and > 25 kg^2^/m^2^, the skeletal muscle radiodensity (SMD) is <41 HU and < 33 HU, respectively.^20^

### 2.3 Statistical analysis

We analyzed the retrieved data with SPSS version 25 (IBM); a *p*‐value of 0.05 was considered statistically significant. For categorical data, results were summarized using counts (n) and percentages (%), with comparisons done using the Chi‐square test. The connection between clinicopathological variables, demographic information, and 12‐month survival was studied using univariate and multivariate binary logistic regression. The 95% confidence interval (CI) and odds ratio (OR) were calculated. The link between ECOG‐PS and hypoalbuminemia with 12‐month survival in patients with advanced NSCLC taking Nivolumab was studied using a Kaplan–Meier curve and a log‐rank test. To find determinants of 12‐month survival, the Cox regression model was applied. The researchers provided hazard ratios (HR), 95% confidence intervals (CI), and *p*‐values. Variable by variable, missing data were removed from the study.

## 3 RESULTS

**FIGURE 1:**
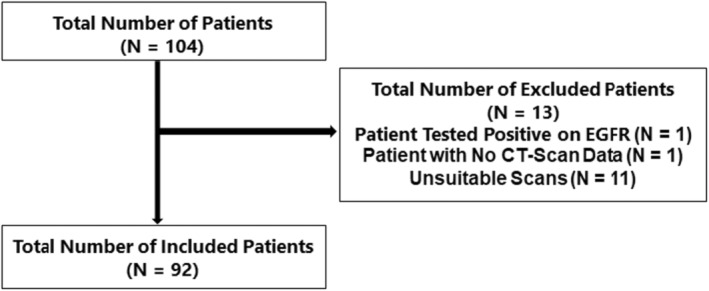
Patients with advanced NSCLC and receiving Nivolumab in second‐line treatment were included in the present study.

The final trial included only 92 of the 104 patients with NSCLC who received Nivolumab as a second‐line therapy **(**Figure 1 **)**.

The clinicopathological characteristics of the patients enrolled are shown in Table 1. Chemotherapy was given to all patients as a first‐line treatment in various combinations. 51% (*n* = 47) of the patients were above the age of 65, and 53% (*n* = 49) were female. Squamous cell carcinoma impacted 40% (*n* = 37) of the patients, whereas adenocarcinoma involved 53% (*n* = 49). 44% (*n* = 40) of patients had hypoalbuminemia, and 70% (*n* = 64) had an NLR greater than 3. 54% (*n* = 50) of the patients had a high VFA, and 70% (*n* = 64) had a high SFI. Low SMI (*n* = 61) and SMD (*n* = 54) were found in 66% (*n* = 61) and 59% (*n* = 54) of the patients, respectively. The median overall survival (OS) after initiating Nivolumab was 15 months.

**TABLE 1:** Clinicopathological characteristics of included patients (*N* = 92).

Demographics	Counts and Percentages (%)
Age (Years)	
<65	45 (49)
65–74	33 (36)
>74	14 (15)
Sex	
Male	43 (47)
Female	49 (53)
Histology	
Adenocarcinoma	49 (53)
Squamous cell carcinoma	37 (40)
Other/Unknown	6 (7)
Eastern Cooperative Oncology Group‐Performance Status (ECOG‐PS)	
0	21 (23)
1	58 (63)
>2	13 (14)
Neutrophil to lymphocyte ratio (NLR)	
<3	28 (30)
3–5	25 (27)
>5	39 (42)
Albumin	
>35 g/L	52 (56)
<35 g/L	40 (44)
Body mass index (BMI) (kg/m^2^)	
<20	15 (16.3)
20–24.9	35 (38)
25–29	26 (28.3)
>30	16 (17.4)
High subcutaneous fat index (SFI)	
No	28 (30)
Yes	64 (70)
High visceral fat area (VFA)	
No	42 (46)
Yes	50 (54)
Low skeletal muscle index (SMI)	
No	31 (34)
Yes	61 (66)
Low skeletal muscle radiodensity (SMD)	
No	38 (41)
Yes	54 (59)
12‐Month survival	
Yes	36 (39)
No	56 (61)

The association between systemic inflammation, CT‐derived body composition measures, clinicopathological features, and 12‐month survival in patients with advanced NSCLC receiving nivolumab as a second‐line treatment is shown in Table 2. At the 12‐month follow‐up, the total number of alive patients had dropped to 36, while the number of deceased had risen to 56. In both the living (63%) and deceased (64%) groups, ECOG‐PS of “1” was substantially higher than ECOG‐PS of “0” or “>2” (*p*‐value = 0.015). Hypoalbuminemia was substantially greater (55%) in the deceased group (*p*‐value = 0.015; Table 2).

**TABLE 2:** The relationship between clinicopathological variables, systemic inflammation, CT‐body composition measurements, and 12‐month survival in patients with advanced NSCLC receiving second‐line Nivolumab therapy.

Variables	All (*N* = 92)	Alive (*N* = 36)	Dead (*N* = 56)	*p*‐Value
Age (Years)				0.799
<65	45 (49)	18 (50)	27 (48)	
65–74	33 (36)	13 (36)	20 (63)	
>74	14 (15)	5 (14)	9 (16)	
Sex				0.725
Male	43 (47)	16 (44)	27 (48)	
Female	49 (53)	20 (56)	29 (52)	
Histology				0.330
Adenocarcinoma	49 (53)	17 (47)	32 (57)	
Squamous	37 (40)	16 (45)	1 (38)	
Others	6 (7)	3 (8)	3 (5)	
Eastern Cooperative Oncology Group‐Performance Status (ECOG‐PS)				0.015
0	21 (23)	12 (33)	9 (16)	
1	58 (63)	22 (61)	36 (64)	
>2	13 (14)	2 (6)	11 (20)	
Neutrophil to Lymphocyte Ratio (NLR)				0.113
<3	28 (30)	14 (50)	14 (50)	
3–5	25 (27)	10 (40)	15 (60)	
>5	39 (42)	12 (31)	27 (69)	
Albumin				0.015
>35 g/L	52 (57)	27 (75)	25 (45)	
<35 g/L	40 (43)	9 (25)	31 (55)	
Body Mass Index (BMI) (kg/m^2^)				0.356
<20	15 (16)	5 (14)	10 (18)	
20–24.9	35 (38)	14 (39)	21 (38)	
25–29	26 (28)	8 (22)	18 (32)	
>30	16 (17)	9 (25)	7 (12)	
High Subcutaneous Fat Index (SFI)				0.659
No	28 (30)	10 (28)	18 (32)	
Yes	64 (70)	26 (72)	38 (68)	
High Visceral Fat Area (VFA)				0.143
No	42 (46)	13 (36)	29 (52)	
Yes	50 (54)	23 (64)	27 (48)	
Low Skeletal Muscle Index (SMI)				0.401
No	31 (34)	14 (39)	17 (30)	
Yes	61 (66)	22 (61)	39 (70)	
Low Skeletal Muscle Radiodensity (SMD)				0.420
No	38 (41)	13 (36)	25 (45)	
Yes	54 (59)	23 (64)	31 (55)	

ECOG‐PS and hypoalbuminemia were significant predictors of 12‐month survival in patients with advanced NSCLC receiving Nivolumab treatment, according to Cox regression (*p*‐value = 0.047 and 0.014, respectively; Table 3).

**TABLE 3:** Cox Regression between clinicopathological variables, systemic inflammation, CT‐body composition measurements, and 12‐month survival in patients with advanced NSCLC receiving second‐line Nivolumab therapy.

Variables		HR	95% CI		*p*‐Value
			Lower	Upper	
Age (years)	<65	Ref			
	65–74	0.618	0.231	1.657	0.339
	>75	1.169	0.361	3.786	0.794
Age		0.976	0.933	1.022	0.302
Sex	Male	Ref			
	Female	1.025	0.374	2.809	0.961
Histology	Adenocarcinoma	Ref			
	Squamous	2.324	0.989	5.460	0.053
	Others	1.933	0.399	9.377	0.413
Eastern Cooperative Oncology Group‐Performance Status (ECOG‐PS)	0	Ref			
	1	0.407	0.167	0.989	0.047
	2	0.178	0.031	1.036	0.055
Neutrophil to lymphocyte ratio (NLR)	<3	Ref			
	3–5	1.507	0.493	4.608	0.472
	>5	1.145	0.389	3.368	0.805
NLR		0.939	0.848	1.041	0.230
Albumin	>35 g/L	Ref			
	<35 g/L	0.291	0.109	0.777	0.014
Hypoalbuminemia		1.062	0.993	1.136	0.080
Body Mass Index (BMI)	<20	Ref			
	20–24.9	1.878	0.530	6.651	0.329
	25–29	1.082	0.221	5.288	0.922
	>30	2.408	0.387	14.991	0.346
BMI		1.043	0.953	1.141	0.359
High Subcutaneous Fat Index (SFI)	No	Ref			
	Yes	0.504	0.112	2.271	0.373
SFI		0.997	0.986	1.009	0.655
High Visceral Fat Area (VFA)	No	Ref			
	Yes	1.765	0.519	6.002	0.363
VFA		1.013	0.993	1.033	0.200
Low Skeletal Muscle Index (SMI)	No	Ref			
	Yes	0.735	0.329	1.642	0.453
SMI		0.980	0.934	1.028	0.410
Low Skeletal Muscle Radiodensity (SMD)	No	Ref			
	Yes	0.492	0.182	1.332	0.163
SMD		1.014	0.960	1.072	0.608

**FIGURE 2:**
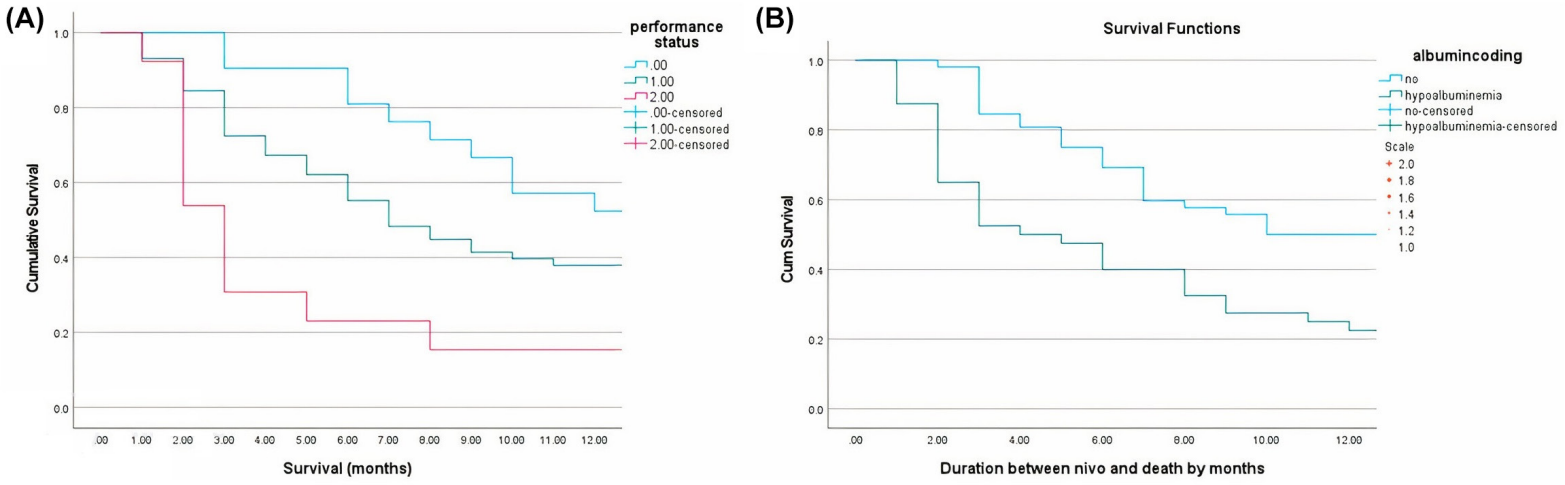
(A) The relationship between ECOG‐PS and 12‐month survival in patients with advanced NSCLC receiving Nivolumab; (B) The relationship between hypoalbuminemia and 12‐month survival in patients with advanced NSCLC receiving Nivolumab.

The Kaplan–Meier curves in Figure 2A,B show the relationship between ECOG‐performance status, hypoalbuminemia, and 12‐month survival in patients with advanced NSCLC on second‐line Nivolumab treatment.

The connection between ECOG‐PS and CT‐derived body composition in patients with advanced non‐small cell lung cancer (NSCLC) receiving nivolumab as second‐line treatment is shown in Table 4. SFI was substantially linked with the ECOG‐PS categories (*p*‐value = 0.042). Low SMD, SMI, and high VFA, on the other hand, were not linked with ECOG‐PS categories (*p*‐values = 0.808, 0.053, and 0.47, respectively). Hypoalbuminemia was substantially linked with ECOG‐PS categories “0” and “1” (*p*‐value = 0.001; Table 4).

**TABLE 4:** The relationship between ECOG‐PS, CT‐body composition, and hypoalbuminemia in patients with advanced NSCLC receiving Nivolumab therapy.

Body Composition	PS 0	PS 1	PS 2	*p*‐Value
Body Mass Index (BMI) (kg/m^2^)				0.445
<20	3 (14)	10 (17)	2 (15)	
20–24.9	7 (33)	23 (40)	5 (38.5)	
25–29	6 (29)	15 (26)	5 (38.5)	
>30	5 (24)	10 (17)	1 (8)	
High Subcutaneous Fat Index (SFI)				0.042
No	2 (7)	21 (75)	5 (18)	
Yes	19 (30)	37 (58)	8 (12)	
High Visceral Fat Area (VFA)				0.47
No	10 (24)	28 (67)	4 (9)	
Yes	11 (22)	30 (60)	9 (18)	
Low Skeletal Muscle Index (SMI)				0.053
No	10 (32)	19 (61)	2 (7)	
Yes	11 (18)	39 (64)	11 (18)	
Skeletal Muscle Radiodensity (SMD)				0.808
No	8 (21)	26 (68)	4 (11)	
Yes	13 (24)	32 (59)	9 (17)	
Albumin				0.001
>35 g/L	17 (81)	32 (55)	3 (23)	
<35 g/L	4 (19)	26 (45)	10 (77)	

## 4 DISCUSSION

To the best of our knowledge, this is the first research to look at the association between systemic inflammation, CT‐BC, pre‐treatment clinicopathological features, and survival in NSCLC patients taking nivolumab for palliative care. Hypoalbuminemia was substantially correlated with survival in the current group, independent of ECOG‐PS and body composition, which was particularly interesting and maybe instructive in using Nivolumab in NSCLC patients.^21^

The current study's findings are in line with a recent review by Tomasik and colleagues of 26,442 patients with advanced NSCLC from 67 studies that reported patients with poor performance status were twice as likely to benefit from ICI as patients with a performance status of 0–1. Furthermore, the present results are consistent with previous studies demonstrating that systemic inflammatory response markers, regardless of ECOG‐PS, have prognostic importance in patients with advanced cancer.^22^ Because ECOG‐PS records the patient's physiological reserve and the systemic inflammatory response catches the catabolic effect on the physiological reserve, the combination of ECOG‐PS and the systemic inflammatory response may have predictive value. Despite the fact that sarcopenia has been linked to poor outcomes in cancer treatment,^23^, ^24^ Tenuta and colleagues found no correlation between sarcopenia and survival in 47 patients with advanced non‐small cell lung cancer (NSCLC) who underwent ICI and used dual‐energy X‐ray absorptiometry (DXA) for body composition measurement. They found that whereas overall survival was unaffected, sarcopenia was linked to a shorter progression‐free survival PFS.^8^, ^25^

As a result, it is uncertain if body composition assessments increase the predictive value of ECOG‐PS and systemic inflammation. A recent study by Hacker and colleagues found that, when compared to sarcopenia, the tumor‐associated systemic inflammatory response was the strongest predictor of prognosis in the phase III EXPAND trial involving good‐performance status patients with gastro‐esophageal cancer. Furthermore, there was no clear link between sarcopenia and survival. As a result, further study should be conducted on the therapeutic targeting of systemic inflammation as a possible method of improving sarcopenia as well as the effectiveness and tolerability of cancer treatment.^26^

The current investigation demonstrated no statistically significant relationship between NLR and 12‐month survival; this conclusion contradicted a previous study that found a link between elevated NLR and poor progression‐free survival (PFS),^27^ quicker time to treatment failure, and overall survival (OS).^9^, ^25^ Similarly, Pavan and colleagues demonstrated that the platelet/lymphocyte ratio (PLR) and normalized lymphocyte ratio (NLR) predicted the occurrence of immune‐related adverse events (irAEs) in 184 patients with advanced NSCLC who received ICI (pembrolizumab, nivolumab, or atezolizumab) as second‐line therapy.^28^ Furthermore, some study indicates that irAEs are associated with poor survival outcomes, while other research indicates that irASs produce a long‐term, sustained disease response in NSCLC patients taking nivolumab.^29^, ^30^ Karayama and colleagues found that increased GNRI, which was calculated from body weight and serum albumin, was associated with better PFS and OS in patients with NSCLC who received nivolumab, regardless of tumor PD‐L1 expression or ECOG‐PS. As a result, albumin may be effective in predicting ICI efficacy.^31^

Because of the small sample size and retrospective study design, the current study had limitations. It is particularly difficult to extrapolate these findings to current clinical practice because immunotherapies are increasingly being utilized as first‐line therapy, and systemic medicine used in the second‐line treatment of NSCLC is no longer considered standard of care. The current study, on the other hand, provides therapeutically relevant information on the link between systemic inflammation and body composition as prognostic factors in NSCLC patients. Prospective research is required to validate prognostic variables for immunotherapy.

## 5 CONCLUSION

Baseline high ECOG‐PS and hypoalbuminemia were linked with poor survival in patients with advanced NSCLC receiving nivolumab as a second‐line therapy. In addition to ECOG‐PS, hypoalbuminemia measurements may be beneficial in predicting clinical outcomes.

## AUTHOR CONTRIBUTIONS

**Randa Saeed:** Conceptualization (supporting); data curation (equal); formal analysis (lead); investigation (lead); methodology (lead); project administration (equal); resources (equal); software (equal); validation (equal); visualization (equal); writing – original draft (lead); writing – review and editing (lead). **Josh McGovern:** Conceptualization (lead); data curation (equal); formal analysis (equal); project administration (lead); writing – review and editing (equal). **Hugo Bench:** Data curation (lead); writing – review and editing (equal). **Ross D. Dolan:** Writing – review and editing (equal). **Donald C. McMillan:** Conceptualization (equal); supervision (lead); writing – review and editing (equal). **Almudena Cascales:** Conceptualization (lead); writing – review and editing (equal).

## FUNDING INFORMATION

This research was not funded by any commercial or profit‐making organization.

## CONFLICT OF INTEREST STATEMENT

The authors state that they do not have any competing interests.

## ETHICS STATEMENT

Because the study involved the monitoring of human subjects, participants provided informed consent. The study was approved by the Caldicott Guardian Ethical Committee.

## CONSENT FOR PUBLICATION

Because the study involved the monitoring of human individuals, informed consent was acquired.

## Data Availability

The datasets used and/or analyzed during the current investigation are accessible upon reasonable request from the corresponding author. The data mentioned in the publication, code book, and analytic code will be freely available to the public at www.uog.co.uk/dataset.
